# Non-ossifying fibromas and fibrous cortical defects around the knee - an epidemiologic survey in a Japanese pediatric population

**DOI:** 10.1186/s12891-022-05330-9

**Published:** 2022-04-22

**Authors:** Makoto Emori, Hiroyuki Tsuchie, Atsushi Teramoto, Junya Shimizu, Emi Mizushima, Yasutaka Murahashi, Hiroyuki Nagasawa, Naohisa Miyakoshi, Toshihiko Yamashita

**Affiliations:** 1 Department of Orthopedic Surgery, Sapporo Medical University School of Medicine, West 16, South 1, Chuo- ku, Sapporo, Hokkaido, 060-8543 Japan; 2 Department of Orthopedic Surgery, Akita University School of Medicine, Akita, Akita, 010-8543 Japan

**Keywords:** Non-ossifying Fibroma, Fibrous Cortical Defect, Prevalence, Pain

## Abstract

**Background:**

The aim of the present study was to evaluate the prevalence of non-ossifying fibroma (NOF) and fibrous cortical defect (FCD) in a Japanese pediatric population and the association between the lesion size and pain.

**Methods:**

This retrospective study, conducted across 10 Japanese institutions, included patients aged 5–15 years who had undergone standard antero-posterior and lateral view radiography of the knee. Using these radiographs, we diagnosed the lesion as a NOF or FCD. Patient demographics, including age, sex, the size and location of the NOF, and chief complaint were recorded. The lesion size was determined using radiographs. Student’s t-test was used to compare the associations between the lesion size and spontaneous pain.

**Results:**

A total of 6222 subjects (3567 boys and 2455 girls) were included in this study. The number of NOF and FCD cases was 143 and 437, respectively, and the prevalence of NOF and FCD was 2.3% and 7.0%, respectively. The average size of NOF and FCD was 22.1 mm (range: 4–102 mm) and 13.2 mm (range: 5–21 mm), respectively. Three patients (2.1%) had pathological fractures due to NOF. Of the 140 NOFs and 437 FCDs, we obtained complaints from the medical records of 126 and 393 patients, respectively. The number of patients with spontaneous pain or other problems with NOF was 68 (54%) and 58 (46%), respectively, that of patients with FCD was 195 (50%) and 198 (50%) patients, respectively. The lesion size was not associated with spontaneous pain in either lesion (*p* = 0.67 and *p* = 0.27, respectively).

**Conclusion:**

The prevalence of NOF and FCD around the knee was lower than that reported in previous studies. The prevalence of NOF increased and that of FCD decreased with advancing age. In both lesions, the lesion size may not be associated with pain.

## Introduction

Non-ossifying fibroma (NOF) and fibrous cortical defect (FCD) are common bone lesions that are usually found in skeletally immature patients aged < 15 years [1]. These are usually asymptomatic and often discovered incidentally. These are the most common lesions that are referred for consultation to orthopedic oncology clinics [2]. Their radiographic features are usually so characteristic that if detected as an incidental finding, a biopsy is not required. The two lesions have different imaging characteristics; FCDs are small lesions primarily located in the bone cortex, whereas larger NOFs are located eccentrically in the medullary cavity [1, 3, 4]. NOFs are large and often symptomatic, while FCDs are small and asymptomatic [3].

NOF and FCD are estimated to be present in up to 30% of children during their skeletal growth period [1, 5]. However, it is difficult to draw definitive prevalence because these rates have been determined from previous studies, which were retrospective case series reported from orthopedic oncology clinics, and these two lesions tend to disappear with age. Larger lesions may cause pain [1]. However, the association between the lesion size and pain is not well known.

Over the years, we have noticed that the prevalence of NOF and FCD is lower than that reported in previous studies. We planned to examine a large group of subjects who had undergone radiography of the knee. We hypothesized that the prevalence of NOF and FCD is lower than that reported in previous studies and larger lesions may cause pain.

The aim of the present study was to evaluate the prevalence of NOF and FCD around the knee in Japanese pediatric patients who underwent plain radiography and to determine the relationship between the lesion size and pain.

## Materials and methods

### Patient selection

This retrospective study was conducted across Kita Akita Municipal Hospital, Sapporo Medical University Hospital, and Sapporo Medical University Orthopedics Affiliated Institutions in Japan (Hakodate Goryoukaku Hospital, Shiroishi Orthopedics Clinic, Takikawa Municipal Hospital, Otaru Saiseikai Hospital, Sapporo Kiyota Orthopedic Hospital, Obihiro Kyokai Hospital, Asabu Orthopedic Hospital, and Sapporo Maruyama Orthopaedic Hospital). Each institution obtained approval for this study from their local research ethics board and then reviewed their database retrospectively. In each institution, radiography was undertaken for all subjects at consultation. The inclusion criteria were (1) patient age: 5–15 years, between April 2010 and February 2021, and (2) patients who underwent standard antero-posterior and lateral view radiography of the knee.

### Non-ossifying fibromas and fibrous cortical defects

**Fig. 1:**
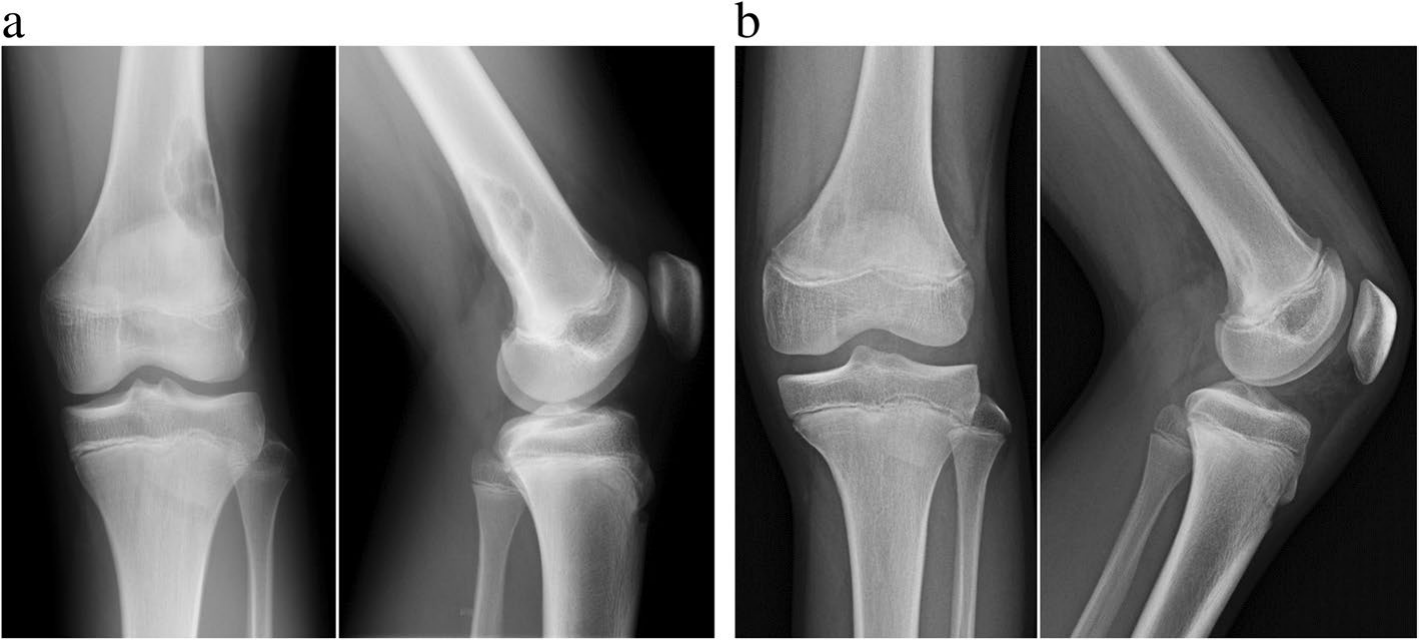
**a** Representative radiograph of a non-ossifying fibroma. 14-year-old boy with non-ossifying fibroma involving the distal posteolateral portion of the femur. Anteroposterior (left) and lateral (right) radiograph show a well-circumscribed radiolucent lesion involving cortex and medullary bone with lobulated thin sclerotic rim. **b** Representative radiograph of a fibrous cortical defect. 14-year-old boy with cortical fibrous defect involving the posteomedial cortex of the femur. Anteroposterior (left) and lateral (right) radiograph show an oval, sharply marginated, radiolucent zone in the cortex surrounded by a rim of sclerotic bone

NOFs and FCDs are benign, well-circumscribed radiolucent lesions in the metaphyseal portions of long bones. The lesions are most commonly located in the distal femur, followed by the proximal and distal tibia and proximal humerus [3]. In most cases, the diagnosis can be made definitively based on characteristic radiographic findings in plain radiographs [6]. We diagnosed the lesion as NOF if it had an oval, rounded or polycyclic, sharply marginated, intramedullary radiolucency in the metaphyseal portion of the distal femur or proximal tibia and fibula, with or without a rim of sclerotic bone (Fig. 1A). We diagnosed the lesion as FCD if it was oval or rounded, sharply marginated, with eccentric radiolucent zones in the medial metaphyseal cortex of the distal femur (Fig. 1B).

### Data collection

Patient demographics, including age, sex, and chief complaint, were recorded. The lesion size was determined on plain radiographs. NOFs were categorized according to the Ritschl classification [7]: Stage A: Eccentric lesion in the cortex near the epiphyseal endplate, which is small, oval to slightly polycyclic in shape, without a sclerotic border; Stage B: Lesions at a variable distance from the epiphysis, with a polycyclic shape, thin but clearly sclerotic borders, and a thin cortex that occasionally protrudes above the surface in an hourglass shape, with no periosteal reaction; Stage C: Lesions with properties similar to those of stage B but also exhibiting increasing sclerosis, which typically starts from the diaphyseal side; and Stage D: Complete homogeneous sclerosis of the lesion.

The chief complaints were classified into two categories: 1) spontaneous pain in the knee area and 2) other problems, such as injury, other bone tumors, or laceration, implying that the lesion was discovered on radiography being performed for another problem for which the patient had visited the doctor. Pain was evaluated by its presence or absence.

### Statistical analyses

Statistical analyses were performed using SPSS version 25.0 (IBM Corp., Armonk, NY, USA). To investigate the association between lesion size and spontaneous pain, the differences between the two categories were assessed using the Student’s t-test; statistical significance was set at *p* < 0.05.

## Results

### Patient demographics and characteristics

A total of 6222 subjects (3567 boys and 2455 girls) were included, and the number of participants is summarized according to age and sex in Table 1. The mean patient age was 11.6 years. The number of patients aged 13 years was the largest, while that of those aged 5 years was the least. The average sizes of NOF and FCD were 22.1 mm (range: 4–102 mm) and 13.2 mm (range: 5–21 mm), respectively. The size of NOF was larger than that of FCD (*p* < 0.0001).

**Table 1:** The number of participants according to age and sex

Age (years)	Total	Boys	Girls
5	198	115	83
6	205	118	87
7	224	135	89
8	276	157	119
9	361	211	150
10	541	327	214
11	728	461	267
12	966	646	320
13	1106	666	440
14	954	521	433
15	663	410	253

### Distribution of NOF and FCD between ages 5 and 15

**Fig. 2:**
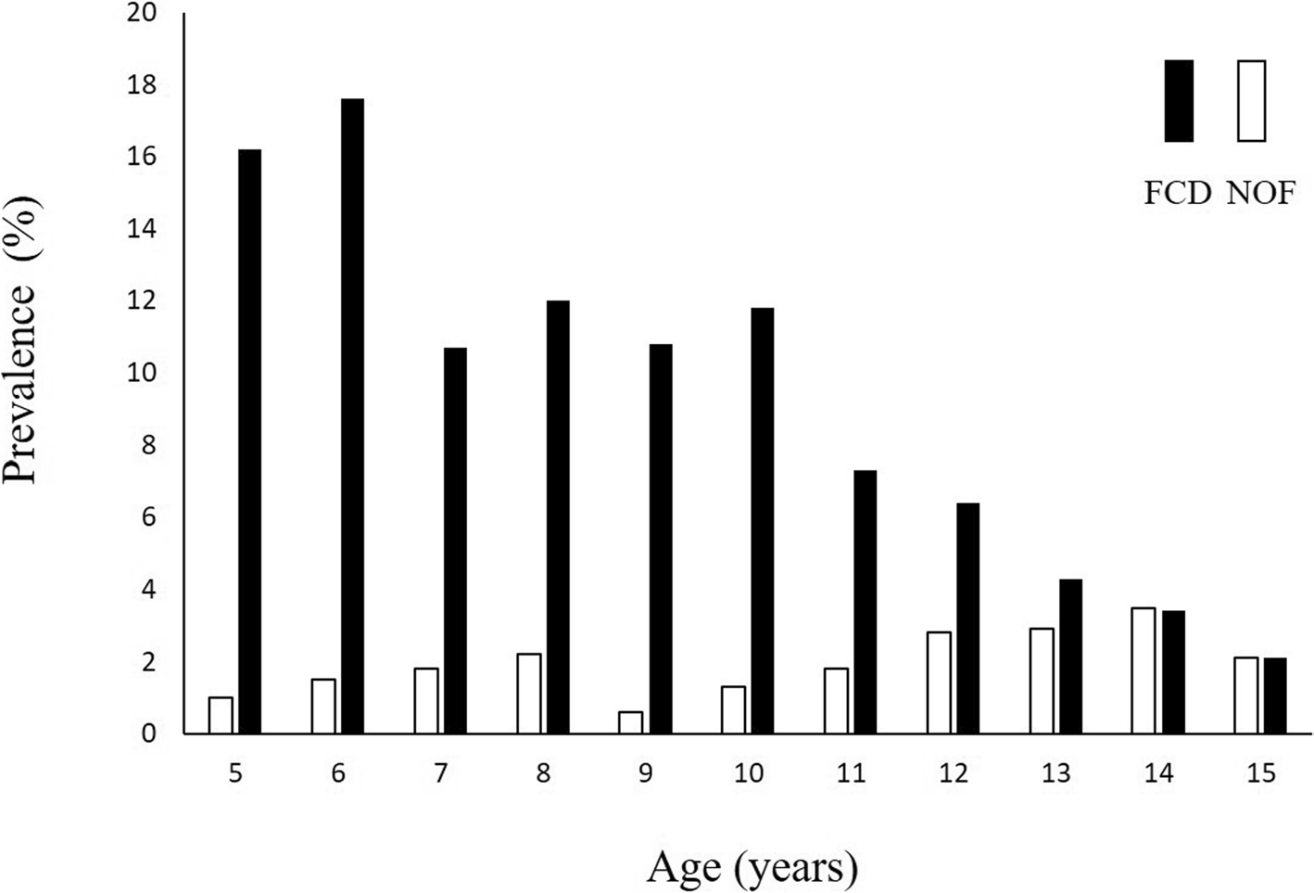
The prevalence of non-ossifying fibroma and fibrous cortical defect according to age

There were 143 and 437 cases of NOF and FCD, respectively, with the prevalence being 2.3% and 7.0%, respectively. Sex, location, and age distribution of NOF and FCD are shown in Table 2. The distal femur was the most common location in NOF. The gender prevalence of NOF was 2.6% in boys and 2.1% in girls and that of FCD was 6.6% in boys and 7.7% in girls. The distribution of NOFs and FCDs according to sex and age are shown in Table 3 and 4. Bilateral NOFs and FCDs were identified in 5 (3.4%) and 83 patients (19%), respectively. The prevalence of NOF and FCD according to age is shown in Fig. 2, and the highest prevalence was seen at ages 14 and 6, respectively. *Ritschl* classifications A, B, C, and D were found in 95, 39, 9, and 0 patients, respectively. Three patients (2.1%) had pathological fractures due to NOF. All cases were classified as stage B and located in the distal femur. The sizes of lesion were 24, 26, and 45 mm.

**Table 2:** Distribution of non-ossifying fibromas and fibrous cortical defects according to sex, location, and age

	NOF		FCD	
	Number	%	Number	%
**Total**	143		437	
**Gender**				
Boys	92	64.3	247	56.5
Girls	51	35.7	190	43.5
**Location**				
Femur	82	57.3	437	100
Tibia	59	41.3		
Fibula	2	1.4		
**Age**				
5	2	1	32	16.2
6	3	1.5	36	17.6
7	4	1.8	24	10.7
8	6	2.2	33	12
9	2	0.6	39	10.8
10	7	1.3	64	11.8
11	13	1.8	53	7.3
12	27	2.8	62	6.4
13	32	2.9	48	4.3
14	33	3.5	32	3.4
15	14	2.1	14	2.1

**Table 3:** Distribution of non-ossifying fibromas according to age and sex

	Boys		Girls	
Age (years)	n	%	n	%
5	1	0.87	1	1.20
6	1	0.85	1	1.15
7	2	1.48	2	2.25
8	3	1.91	3	2.52
9	2	0.95	0	0.00
10	2	0.61	5	2.34
11	7	1.52	6	2.25
12	18	2.79	9	2.81
13	23	3.45	9	2.05
14	21	4.03	12	2.77
15	12	2.93	3	1.19

**Table 4:** Distribution of fibrous cortical defects according to age and sex

	Boys		Girls	
Age (years)	n	%	n	%
5	17	14.78	15	18.07
6	28	23.73	8	9.20
7	16	11.85	8	8.99
8	17	10.83	16	13.45
9	26	12.32	13	8.67
10	30	9.17	38	17.76
11	22	4.77	31	11.61
12	36	5.57	26	8.13
13	23	3.45	25	5.68
14	21	4.03	11	2.54
15	11	2.68	3	1.19

### Relationship between lesion size and patient’s complaint

For the determination of this relationship, we excluded patients with pathological fractures. Of the 140 NOFs and 437 FCDs, we obtained complaints from the medical records of 126 and 393 patients, respectively. The number of patients with spontaneous pain or other problems with NOF was 68 (54%) and 58 (46%), respectively, that of patients with FCD was 195 (50%) and 198 (50%) patients, respectively. The lesion size was not significantly associated with spontaneous pain in either NOF or FCD (*p* = 0.67 and *p* = 0.27, respectively).

## Discussion

To our knowledge, this retrospective study is the largest study on NOF and FCD. In this study, we evaluated the prevalence NOF and FCD around the knee in Japanese pediatric patients and determined the association between the lesion size and pain. The prevalence of these two lesions was much lower than that reported in previous studies [1, 8]. The prevalence of NOF increased and that of FCD decreased with advancing age. Half of the patients with FCD complained of spontaneous pain. However, the lesion size and spontaneous pain may not be associated.

NOF and FCD, collectively called metaphyseal fibrous defects, have been considered to be synonymous because of similar pathologic findings, although they differ in size and primary locations in the long bones [4, 9]. NOF lesions are principally metaphyseal in location, and the most common sites include the distal femur, proximal tibia, distal tibia, proximal humerus, fibula, and radius are the most common sites [3]. Less than 5% of NOFs are multifocal. Most multifocal NOFs can develop sporadically; however, they also develop in patients with neurofibromatosis type 1 and Jaffe-Campanacci syndrome [1]. Recently, NOF has been considered a neoplasm because of Ras-MAPK activation by somatic mutation [10]. Thus, these two lesions are different entities because of their differing clinical and biological characteristics. FCD maintained the same position [11], while NOF advanced proximally or distally with age. Therefore, FCD probably occurs when tendons are inserted into the perichondrium of the epiphyseal plate [7, 11, 12]. A male predominance was found in the present study, which is consistent with the findings of previous studies [6, 7, 12].

It is estimated that NOF may be present in up to 30% of children [1, 5]. However, this prevalence is not accurate because previous studies were retrospectively cases series. Sontag and Pyle examined the skeletal roentgenograms of healthy children until the age of 18 years and found cystic lesions (consistent with FCD) in 22% of the girls and 53% of the boys [11]. However, no similar research has been conducted on NOF. We only evaluated the patients when they underwent radiography; however, the prevalence of NOF and FCD in this study may be more accurate than that reported previously. No ethnic prevalence has been described [3].

*The Ritschl* classification is based on the clinical course of the healing process. *Ritschl* classification A was the most common type among the patients in our study, probably because our patients were younger than those studied previously [6].

NOF and FCD differ in terms of size and clinical course. The two lesions frequently disappear with advancing age. NOFs are large and often symptomatic, while FCDs are small and asymptomatic [3]. In our study, the chief complaints were classified into two categories: spontaneous pain and other problems. The ratio of spontaneous pain was higher in the NOF group. However, many patients with FCD also had spontaneous pain, regardless of the etiology. Neither of the lesions showed a significant association between pain and the lesion size. In the future, we will evaluate the association between sports activity and FCD.

Pathologic fractures have been reported to occur in up to 20% of NOFs [13]. However, the occurrence of pathologic fractures associated with NOF in this study was lower than that reported previously. NOFs that are more than 33 mm long are likely to cause fracture [9]. However, it is unclear that the lesion size is associated with the occurrence of fracture [14]. While absolute size parameters may be useful in predicting pathologic fracture rate, they do not imply a requirement for prophylactic curettage and bone grafting. Our pathologic fracture cases were all categorized as *Ritschl* classifications B. Patients with a stage B lesion have an increased risk of suffering a pathologic fracture [6]. Thus, the combination of lesion size and *Ritschl* classification may be important for the evaluation of predicting pathologic fracture.

This study has a few limitations. First, X-rays should only be used for pediatric patients only when there is a potential benefit that may assist in the diagnosis of a condition or to monitor any treatment. However, we perform X-ray to assess the following conditions: injury, infection, abnormal bone growths and bone changes seen in metabolic conditions, bone cancer, and the location of foreign objects in soft tissues or in or around bones.

Second, we only evaluated lesions around the knee, although these lesions occur at other sites. Third, the prevalence of NOFs and FCDs was much lower than that reported in previous studies. However, this result may be due to racial differences. Fourth, the cause of pain may not necessarily be NOF or FCD but other disorders such as Osgood–Schlatter disease.

## Conclusion

We evaluated the prevalence of patients with NOF and FCD around the knee and the association between the lesion size and pain. The prevalence of NOF and FCD was 2.3% and 7.0%, respectively, which is much lower than that reported in previous studies. FCDs are small and usually asymptomatic, but a relatively large number of patients complain of pain.

## Data Availability

The datasets supporting the conclusions of this article are included within the article (and its additional files). Additional datasets used for the current study are available from the corresponding author upon request.

## Abbreviations

FCD: Fibrous Cortical Defect
NOF: Non-Ossifying Fibroma
MAPK: Mitogen-Activated Protein Kinase
